# A rapid screening method for evaluating the total migratable hydrocarbons in paper products by headspace gas chromatography

**DOI:** 10.1039/c8ra09055f

**Published:** 2019-04-01

**Authors:** Yi Dai, Chuxing Zhu, Meigui Xue, Xin-Sheng Chai, Chunxia Chen, Runquan Chen, Huichao Hu

**Affiliations:** College of Material Engineering, Fujian Agriculture and Forestry University Fuzhou 350002 China hhc_huichao@163.com +86-15960036871; State Key Laboratory of Pulp and Paper Engineering, South China University of Technology Guangzhou 510641 China; Dongguan Polytechnic Guangdong 523808 China; National Center for Paper Product Quality Supervision and Inspection Dongguan 523808 China

## Abstract

Herein, we report a rapid screening method for evaluating the hydrocarbon contamination in paper samples by headspace gas chromatography (HS-GC). This method was based on conducting the near-complete migratable release of hydrocarbons from a paper matrix to the headspace in 35 min at 98 °C. By programming the GC column temperature, other co-existing volatile organic compounds in the sample can be effectively separated from the migratable hydrocarbons. To simplify the method calibration, the concept of total migratable hydrocarbons was introduced and *n*-pentadecane was used as the standard hydrocarbon compound in the calibration. The results indicate that the present method offers good precision (the relative standard deviation < 9.8%) and accuracy (recovery between 94.3 and 101%). The present method can be a valuable tool for the quality assessment of total migratable hydrocarbons of paper products, aiming at providing a good guidance for safely using the recycled paper-based materials in various applications.

## Abstract 2

Herein, we report a rapid screening method for evaluating the hydrocarbon contamination in paper samples by headspace gas chromatography (HS-GC).

## Graphical abstract

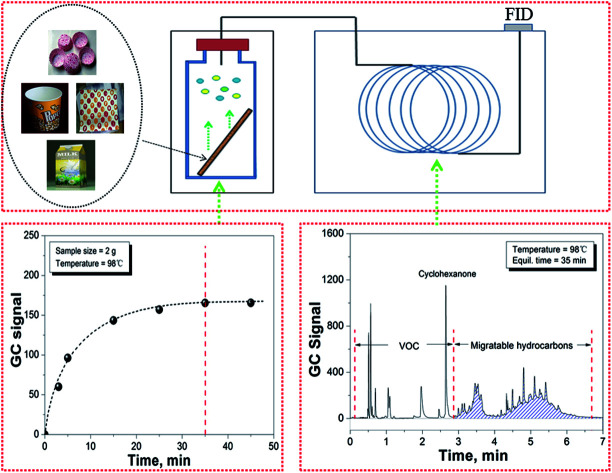

## 1. Introduction

Attributed to the nature of renewability and biodegradability,^1,2^ papers and paperboards have been increasingly used as packaging materials in many applications. The utilization of recycled fibers from waste paper can greatly save the cost for papermaking and also reduce the use of forest resources and thus protect environment. Nowadays, up to 90% of recycled fibers are used in the production of boxboard paper and corrugated based paper for packaging industries.^3^ However, there is a considerable amount of hydrocarbons (mainly the mineral oils (MOs) including both saturated hydrocarbons and aromatic hydrocarbons that originated from the organic solvents for printing inks^4^) present in the recycled fibers of manufactured paper and paperboards. Since these organic substances are toxic,^5^ they could pollute the indoor air or pose potential risk to contaminate foods when such paper products are used for packaging during storage. Therefore, it is important to find an effective method to determine the amount of MOs in the packaging papers, from which a predicting model based on the storage conditions can be developed. Such a model can provide a good guidance for safely using the recycled paper-based packaging materials.

MOs present in printing inks and recycled paperboards are typically a group of volatile or less volatile mixtures of primarily *n*-C_18_–*n*-C_23_ hydrocarbon compounds.^6^ It is essential for the analytical methods to effectively separate the individual MO species from the other co-existing substances in paper samples. In general, the MO species are first transferred from the paper matrix to the liquid medium by solvent extraction, *e.g.*, using hexane/ethanol or hexane/acetone solution for 2 h at room temperature.^6^ In order to shorten the extraction time, a pressurized liquid extraction (PLE) technique was studied,^7^ in which the process was conducted in a vessel under high temperature and pressure. As a result, the extraction time was reduced to only 5 min, but similar extraction yield was maintained as that in the previous method. In the MOs' quantification analysis, the separation-based techniques are usually applied since the compositions of MOs themselves are very complicated. For example, the measurement technique combining high performance liquid chromatography (HPLC) with gas chromatography is typically used in MOs' analyses reported in the literature.^8,9^ Clearly, the separation of not only MOs but also the interferences from co-existing species, such as polyolefin oligomers and the other less- or non-volatile organic compounds extracted from the sample matrix, is a very difficult task in both HPLC and GC analysis. Moreover, this coupled chromatographic system is so expensive that the dedicated instrumentation is only available in a few laboratories. To eliminate the above HPLC step, a solid phase extraction (SPE) pre-treatment procedure before the GC has been proposed, in which a silver silica gel is used as a functional material placed in the SPE cartridge.^10^ However, the preparation of the SPE cartridge is time-consuming since the silica gel must be heated at 400 °C for overnight, mixed silver nitrate and kept in dark for about 12 h, and then heated at 75 °C for overnight to eliminate residual water from the material. Because some MOs have low volatility and some are even non-volatile, the GC measurements can also cause problems such as the contamination of injection port and the column as well as poor repeatability during testing. As matter of fact, the most harmful portion of MOs are the volatile species because they could be migrated from the packaging paper matrix to the surrounding air and then be adsorbed by the packaged stuff (*e.g.*, food) during storage and transportation.^11^ Studies have shown that the absorption of MOs by the human body decreases with the species' molecular size,^4^*i.e.*, if the carbon number of MOs is smaller, the toxicity of MOs will be greater.^12^ Therefore, it is meaningful to find a simple way that can determine the migratable MO species (*i.e.*, with low volatility) from the paper products in the risk assessment. Recently, a method for simulating the MOs migrated from a paperboard was proposed.^13^ In this method, an adsorbent material (Tenax) was used as the simulant placed directly on the tested sample at 40 °C for 10 days and then, the migrated MOs adsorbed by Tenax were extracted by a solvent and analyzed by GC or HPLC. Clearly, this method is not only complicated but also very time-consuming. Moreover, the species adsorbed by Tenax included both MOs and other volatile hydrocarbons. Therefore, the interferences from some co-existing species on the MOs' separation would still be the primary problem in GC or HPLC analysis, as mentioned above.

Headspace-based GC method (HS-GC) is an effective technique to minimize the effect from non-volatile species in the sample matrix.^14^ One of the great advantages of HS-GC analysis is that the sample conditioning (*e.g.*, heated to a desired temperature) can be performed automatically in a headspace oven,^15–17^ which makes the method much simpler and efficient. Moreover, this technique can determine the analyte migrated from the complicated sample without any solvent or solid-phase extraction pre-treatment and thus, it is suitable for quantifying the migratable hydrocarbons (*i.e.*, the low volatile species) in the paper materials.

In this study, we propose a rapid screening method for evaluating the migratable organic contamination from paper materials, based on HS-GC for measuring the total hydrocarbons (including MOs) at the designated conditions. The main goals were to find the GC conditions to separate the low volatile hydrocarbons (mainly MOs) from the high volatile organic compounds (VOCs) present in the samples, the sample size, the temperature and the time used in the headspace equilibration, and simplification of the method calibration in the migratable hydrocarbons testing.

## 2. Materials and methods

### 2.1 Chemical and samples

All chemicals used in the experiment were of analytical grade and purchased from commercial sources. Paper samples used in the investigation were collected from several local paper mills.

### 2.2 Apparatus and operation

HS-GC measurements were carried out using an automated headspace sampler (TriPlus 300, Thermo Fisher) and a GC system (Agilent GC 7890A, US) equipped with a flame ionization detector (FID) and a DB-5 capillary column (30 m × 0.32 mm × 0.25 μm) from J&W Scientific (USA), operating with nitrogen carrier gas (flow rate = 3.8 mL min^−1^). Headspace operating conditions were as follows: (i) pressurization pressure = 1.00 bar, (ii) carrier gas pressure = 1.50 bar, (iii) vial pressurization time = 15 s, (iv) sample loop fill time = 10 s, and (v) transfer time = 20 s. GC measurement conditions were as follows: the oven temperature was maintained for 2 min at initial temperature (35 °C); then, the instrument was programmed at 100 °C min^−1^ from 35 to 200 °C and maintained for 3 min.

### 2.3 Sample preparation and HS-GC measurement

First, one gram of tested paper sample (cut in pieces) was added in a headspace sample vial. Then, the vial was sealed immediately with a PTFE/butyl septum and placed in the headspace sampler for equilibration, followed by GC measurement.

## 3. Results and discussion

### 3.1 GC separation of hydrocarbons from the other VOCs in paper samples

**Fig. 1:**
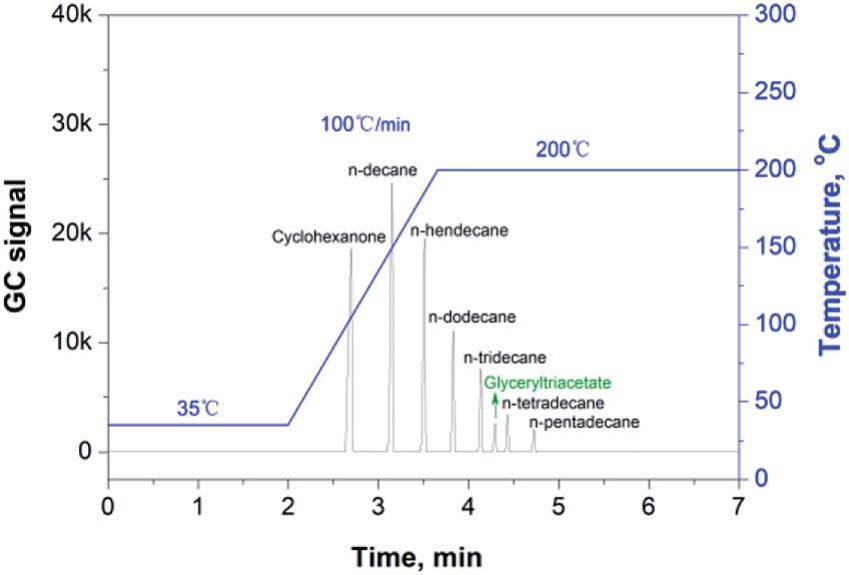
GC separation of cyclohexanone from the migratable hydrocarbons species (*n*-alkanes).

There is a significant amount of recycled fibers used in the production of packaging papers, in which many volatile organic compounds (VOCs), such as methanol produced by the alkaline pulping industry and cyclohexanone introduced by the paper printing industry, also remained in the paper matrix. Therefore, it is particularly important to separate these substances from the migratable hydrocarbons *via* GC analysis. Cyclohexanone, one of the typical organic solvents used in printing, can be regarded as a VOC species with the highest boiling point.^18^ Therefore, we can use the cyclohexanone peak as the indicator in the GC measurement to check if the GC conditions were appropriate to separate the group of VOCs from the group of migratable hydrocarbons (mainly MOs). Fig. 1 shows the chromatogram of cyclohexanone and a number of volatile hydrocarbons (*n*-alkanes) dissolved in glyceryl triacetate (as a blank solvent), and it proves that the given GC conditions are optimal for separating cyclohexanone from the co-existing hydrocarbon species in the paper samples.

### 3.2 Conditions to extract the migratable hydrocarbons from paper sample using headspace

#### 3.2.1 Effect of temperature and time

**Fig. 2:**
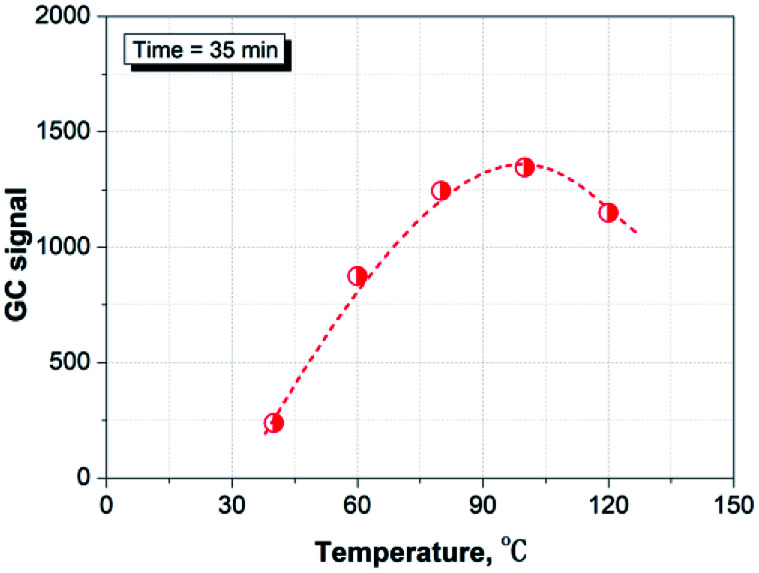
Effect of equilibration temperature on the GC signal for *n*-pentadecane.

Fig. 2 shows the effect of temperature on the release of a migratable MO species (*n*-pentadecane) from a paper sample at a given headspace equilibration time (35 min). The result shows that the signal of a hydrocarbon species released to the headspace increases with the increase in temperature until it reaches 95–100 °C. However, further increase in temperature reduces the signal intensity. We believe that this is caused by the feature of the sampling mode in the given (pressurized) headspace sampler system because the high total pressure in the vial leads to more dilution of the vapor sample.^19^ In the present case, the high vapor pressure at the temperature above 100 °C is mainly caused by water since it can be entrapped in the paper sample (about 9%) due to the high hydrophilicity of cellulose fibers.^20^ To minimize the effect of the water pressure, we chose 98 °C as the suitable temperature for the headspace extraction in the following investigation.

**Fig. 3:**
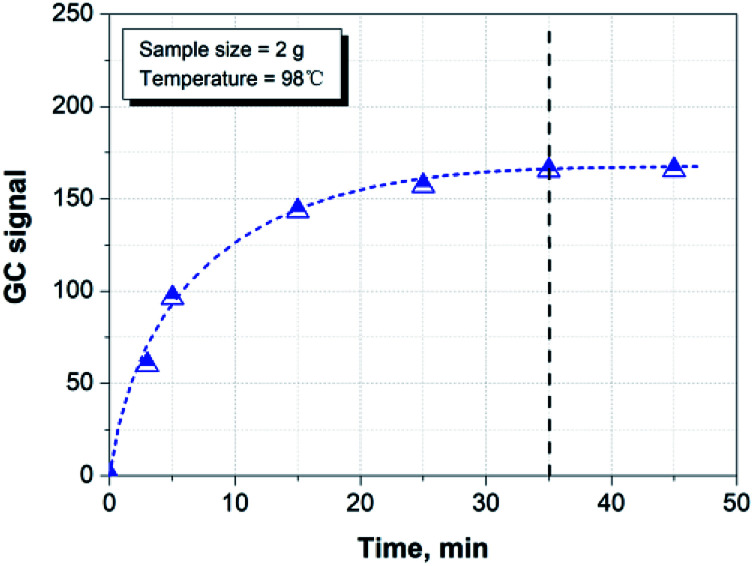
Effect of equilibration time on the GC signal for the hydrocarbon release.

Fig. 3 shows the effect of time on the headspace extraction at 98 °C. It can be seen that the hydrocarbon species released from the paper matrix reaches equilibration after 35 min at the given conditions. Therefore, we chose 35 min as the sample equilibration time for the rest of the study.

#### 3.2.2 Effect of sample size

**Fig. 4:**
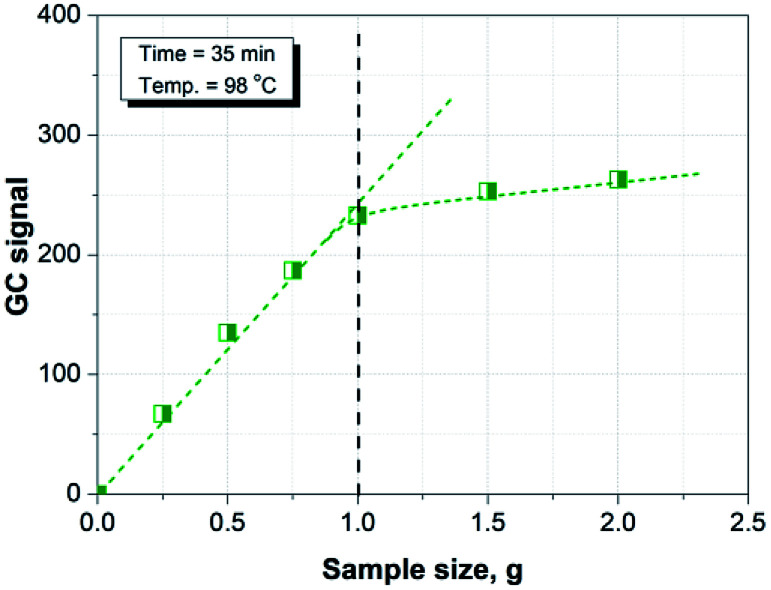
Effect of sample size on the GC signal response.

Due to the very low content of hydrocarbons in the paper samples, a large sample size involved in the testing will be helpful for improving the detection sensitivity in the present method.^14^Fig. 4 shows the effect of paper size on the GC signal in a real sample testing at the given equilibration conditions. It can be seen that the GC signal for the hydrocarbon species almost linearly increases with the increase in sample size. However, it becomes non-liner when the sample size is greater than 1 g. This was caused by the changes in the actual headspace volume in the given vial, which affects the total pressure during the headspace sampling (leads to a more dilution) and thus the results in the GC measurement.^14^ To minimize the pressure effect, we used a fixed sample size in both sample testing and calibration.

### 3.3 Method calibration

**Fig. 5:**
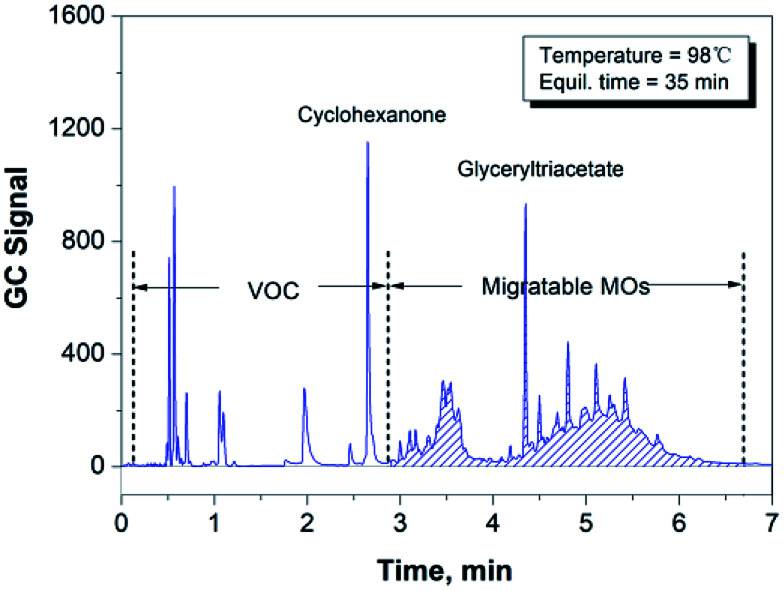
The gas chromatogram of the volatiles of hydrocarbons in the paper.

Fig. 5 shows the chromatogram of the HS-GC measurement for testing a recycled paper sample. It can be seen that the interference of the VOC species presented in the sample is not a problem since they can be well-separated from the migratable hydrocarbons at the given GC conditions. However, the procedures for quantification and method calibration could be very complicated if each of the individual hydrocarbon species must be determined. In practical applications, the total content of migratable hydrocarbons could be a more important index for checking the degree of the hydrocarbon contamination or the performance of hydrocarbon removal-related processes, *e.g.*, the deinking process for recycled papers.^21^ Therefore, we introduced a concept of total migratable hydrocarbons in the present study for the sake of simplicity.

In this work, *n*-pentadecane was used as the model hydrocarbon compound in the calibration, and hence a set of standard *n*-pentadecane samples was prepared in glyceryl triacetate (as the solvent). By using HS-GC for measuring the peak areas of *n*-pentadecane on these samples, the standard calibration curve was obtained, which can be expressed as

1 *A* = 3.99(±0.22) + 753(±13)*m* (*n* = 6, *R*^2^ = 0.999)

where *A* and *m* represent the GC signal peak area of *n*-pentadecane in the vapor phase and the amount of *n*-pentadecane (in μg) added in the headspace sample vial, respectively.

In the real sample testing, all peak areas of each MO appeared to be integrated in the given range of the retention time, as shown in Fig. 5. By the established calibration curve (or eqn (1)), the corresponding content of hydrocarbon in the paper sample, counted as *n*-pentadecane, can be calculated by2
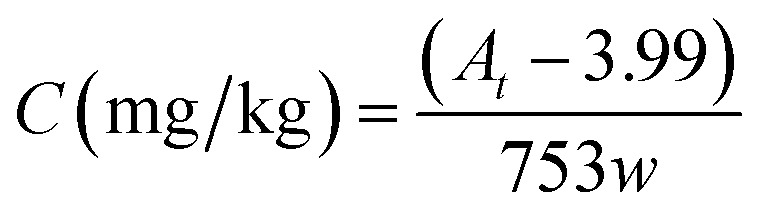
where *A*_*t*_ and *w* represent the total peak area of migratable MOs detected by HS-GC and the paper weight (in g) used in the testing, respectively.

### 3.4 Method evaluation

#### 3.4.1 Method repeatability

The repeatability of the present method was investigated by triplicate tests on four paper samples from the different sources. As listed in Table 1, the relative standard deviation (RSD) in these measurements was less than 9.8%.

**Table 1:** Repeatability test of the method

Replica no.	Signal of migratable hydrocarbons			
	Sample 1	Sample 2	Sample 3	Sample 4
1	289	492	133	323
2	314	471	141	390
3	318	500	143	342
Average	307	488	139	352
RSD/%	5.12	3.07	3.81	9.80

#### 3.4.2 Method validation

In this study, the accuracy of the present method was evaluated by a recovery test, *i.e.*, spiking different amounts (known) of *n*-pentadecane into the real paper samples and checking their actual contents measured by the present method. Table 2 shows the results from the recovery test; *n*-pentadecane recovered by this method ranged from 94.3% to 101%. Therefore, the present method is justifiable to be used in the determination of the migratable hydrocarbons in paper samples.

**Table 2:** Recovery test

Sample no.	*n*-Pentadecane content (mg kg^−1^)		Recovery%
	Added	Measured	
1	14.0	13.3	95.0
2	17.5	17.3	98.9
3	21.0	20.1	95.7
4	24.5	24.7	101
5	31.5	29.7	94.3

#### 3.4.3 Sensitivity of the method

The limit of quantitation (LOQ) for the present method was calculated by the following equation, based on the information shown in eqn (1):3
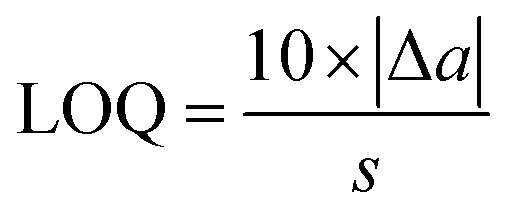
where *a*, Δ*a* and *s* represent the intercept, the uncertainty of the intercept, and the slope in eqn (1), respectively.

Counted as *n*-pentadecane, the LOQ of the present method is 2.9 μg, which corresponds to 2.9 mg kg^−1^ of migratable hydrocarbons in the paper samples. The mass ratio of packaging paper on the packaged food ranges from 1 : 5 to 1 : 25, with an average of around 1 : 10.^6^ Therefore, the LOQ of the present method is sufficient to meet the sensitivity requirements for the safety examination of migratable hydrocarbons in food-related packaging materials.

### 3.5 Application: contents of migratable hydrocarbons in different paper samples

**Fig. 6:**
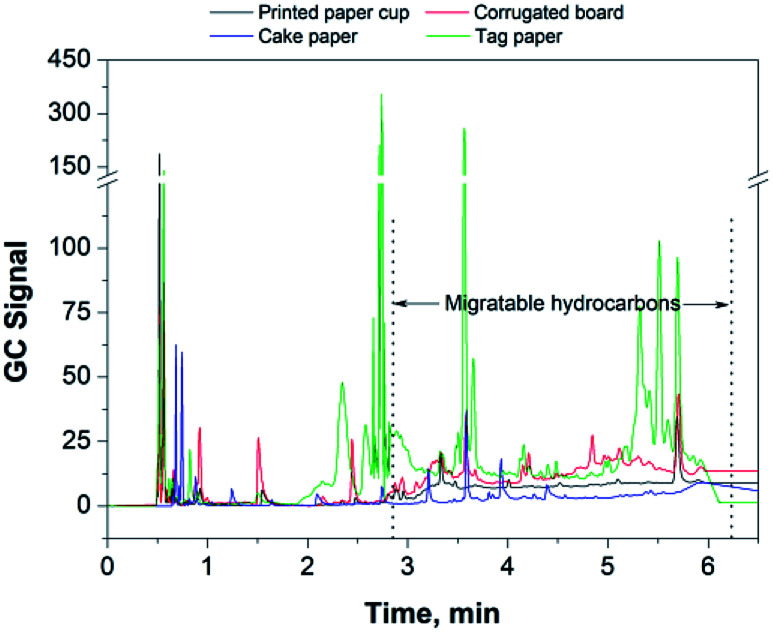
The gas chromatogram of the volatiles of hydrocarbons for some paper materials.

In this study, the content of migratable hydrocarbons (counted as *n*-pentadecane) for different food packaging papers was examined and the results are shown in Table 3 and in Fig. 6. It can be seen that the contents of migratable hydrocarbons is noticeably different in different types of food packaging papers. It can be seen that printing is one of the major sources causing paper pollution through the release of migratable hydrocarbons. There was a significant amount of migratable hydrocarbons remaining in the corrugated board due to the use of recycled fibers. Therefore, considerable attention must be paid on these two aspects, aiming at minimizing the potential contamination by migratable hydrocarbons in food-related packaging materials, especially in those products subjected to a long-term storage.

**Table 3:** Amount of migratable hydrocarbons in different paper sample

Paper type	Specification	Migratable hydrocarbons^a^, mg kg^−1^
Kitchen tissue	No printing	N/A
Milk box	No printing	N/A
	Printed	72.9
Disposable paper cup	No printing	127.3
	Printed	178.9
Snack box	Printed	364.5
Cake paper	Printed	401.8
Corrugated board	No printing	1501.1
Tag paper	Intensely printed and placed in the snack	5007.9

a Counted as *n*-pentadecane; N/A: not detected.

## 4. Conclusions

We have proposed a new and effective method for the determination of total migratable hydrocarbons in paper samples based on the HS-GC measurement. The results show that the present method offers a good precision (the relative standard deviation < 9.8%) and accuracy (recovery between 94.3 and 101%). It is simple, rapid and is suitable to be used for the quality assessment of total migratable hydrocarbons in paper products, thus providing a good guidance for safely using the recycled paper based materials in specific applications, *e.g.*, food packaging and personal care products for children.

## Conflicts of interest

There are no conflicts to declare.

## Supplementary Material
